# LVAs in a Pedicled SIEA Flap for the Treatment of Recurrent Lymphocele of the Groin Using Superficial Veins of the Flap for Lymphovenous Anastomosis: A Case Report and Literature Review

**DOI:** 10.1055/s-0044-1782142

**Published:** 2024-06-13

**Authors:** Federico Facchin, Elmar Fritsche, Alberto Franchi

**Affiliations:** 1 Plastic Surgery Unit, San Bortolo Hospital, Vicenza, Vicenza, Italy; 2 Department of Hand- and Plastic Surgery, Luzerner Kantonsspital, Lucerne, Switzerland

**Keywords:** LVAs, lymphocele, lymphorrhea, lymphovenous bypass

## Abstract

Persistent lymphocele of the groin is a complication of groin surgery that can severely impact the quality of life. The restoration of the interrupted lymphatic pathway is considered by many authors the ideal treatment to prevent a recurrence. However, multiple aspiration procedures and surgical revisions can compromise the availability of local veins needed for a lymphovenular bypass surgery. In addition, surgical debridement of a long-standing lymphocele can generate extensive dead space and contour deformity. A flap delivering additional venules for trans-flap lymphovenular anastomoses (LVAs) can overcome both problems by providing soft tissue and competent veins harvested outside the zone of injury.

A successful case of severe groin lymphocele treated with trans-flap LVAs from an abdominal-based flap is presented. The patient was referred to us for a recurrent lymphocele developed in the right groin after lipoma excision that persisted despite multiple surgical attempts. After the identification of patent and draining inguinal lymphatic vessels, a pinch test was used to design a mini-abdominoplasty superficial inferior epigastric artery flap. The superficial veins of the cranial incision were identified and anastomosed to the lymphatic vessels after the pedicled flap harvested and insetted in the groin.

The early restoration of lymphatic drainage and the optimal aesthetic outcome supports the combined approach offered by trans-flap LVAs as a valuable therapeutic option for severe and persistent lymphocele.

## Introduction

Prolonged drainage output, lymphorrhea, lymphocele, and lymphedema are complications related to the injury of lymphatic vessels and they have been reported after various surgical procedures (from inguinal hernia repair to lymph node dissections and sarcoma resection). 1 Usually temporary, lymphoceles can require conservative management with multiple aspirations of the retained lymph and compression. When persistent they affect the patient's daily life, with an increased risk of recurrent infections and hospitalizations.

Multiple surgical strategies have been developed over the decades: surgical debridement, layered suture, lymphatic vessel embolization, negative pressure wound therapy and macroscopic or microscopic vessel ligation are frequently attempted with varying results. 2 3 4 5 6

For this reason, locoregional or free flaps have been proposed to close the dead space after extensive oncologic resections, lymphadenectomies, radiotherapy, and multiple revision procedures. 7

Nevertheless, lymphatic flow restoration is considered by many authors the ideal approach to physiologically address the aforementioned complications related to the interruption of the lymphatic pathway, limiting the risk of recurrence.

Recent papers have shown that bypass procedures aimed at deviating the lymphatic flow from noncompetent lymphatic vessels to the venous system are effective tools in treating and preventing lymphatic complications. 8 9

The availability of functioning and nonback flowing recipient veins is the prerequisite to performing efficacious lymphovenular anastomosis (LVA). 10 However, multiple surgical procedures, lymphadenectomies, and radiotherapy can damage the local venous system, preventing the feasibility of LVA. In addition, a chronic lymphocele can lead to compression and scarring of surrounding tissue with an increased risk of contour deformity after its removal.

Diverging the lymphatic flow to a venule provided by a flap with a technique described by di Summa et al with the term Lymphatic Flow-Through consists of harvesting a pedicle flap containing additional recipient veins thus allowing the lymphaticovenular bypass to be performed within the flap and dead space obliteration at the same time. The majority of cases presented in the literature with this type of approach are preventive. 11 12

We present a case of a patient suffering from recurrent lymphocele successfully treated with the combination of a pedicled flap based on the SIEA and LVAs performed on recipient veins on the flap. In addition, we review the literature to find all the paper dealing with treatment of recurrent lymphocele with LVAs and flaps.

## Case

**Fig. 1:**
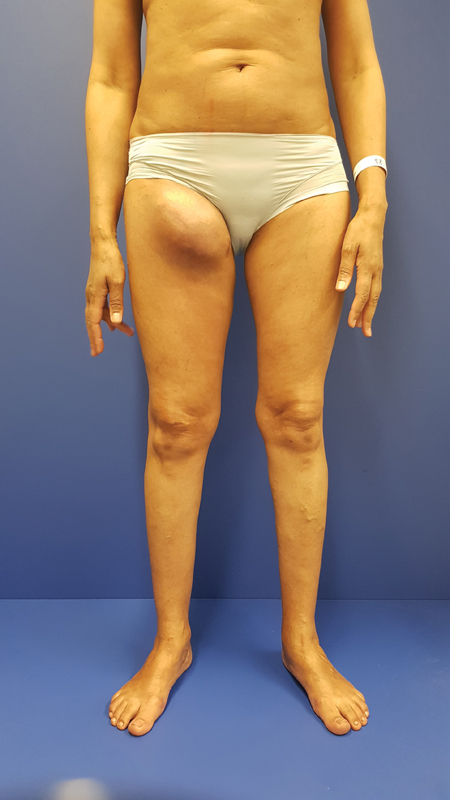
Preoperative image with chronic lymphocele of the right groin.

A 46 year-old-woman presented with a recurrent lymphocele of the right groin developed after lipoma removal 10 months before in another institution ( Fig. 1 ). Before consultation, she underwent conservative treatment with multiple sessions of compression and aspiration and two separate surgical treatment attempts based on surgical debridement, lymphatic vessels ligation.

When the patient was referred to us, she was exhausted by the multiple aspirations (two to four times a week) complaining of discomfort and swelling of the entire limb. She was concerned about additional scarring and the possibility of permanent disfigurement.

A preoperative lymphoscintigraphy study showed lymphatic leakage causing constant refill of the lymphocele. The patient was proposed for lymphatic flow-through LVA superficial inferior epigastric artery (SIEA) flap designed to mimic a mini-abdominoplasty.

**Fig. 2:**
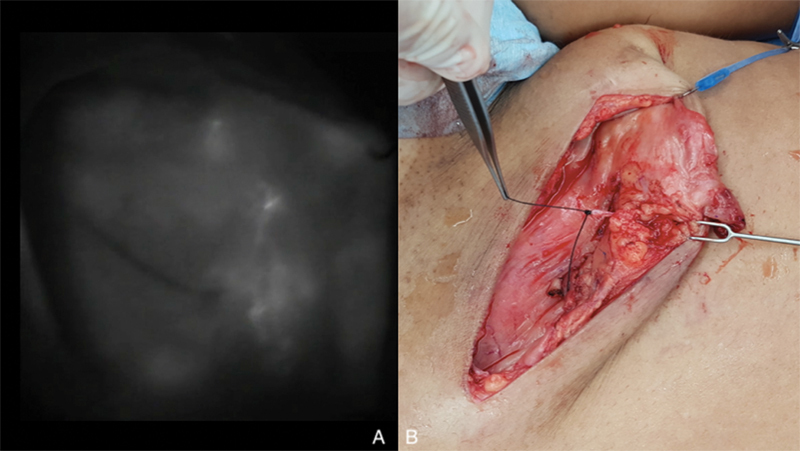
( **A** ) ICG image of three light spots of lymphatic leakage. ( **B** ) Identification of a lymphatic vessel.

After the lymphocele was incised and emptied, indocyanine green (ICG) was injected in the foot (dorsum and medial malleolar area) to find the leakage on the inner surface of the capsule. The three main light spots identified on the inner caudal surface corresponded to the leaking lymphatic vessels. An extensive dead space and the absence of adequate proximal recipient veins were evident ( Fig. 2 ).

**Fig. 3:**
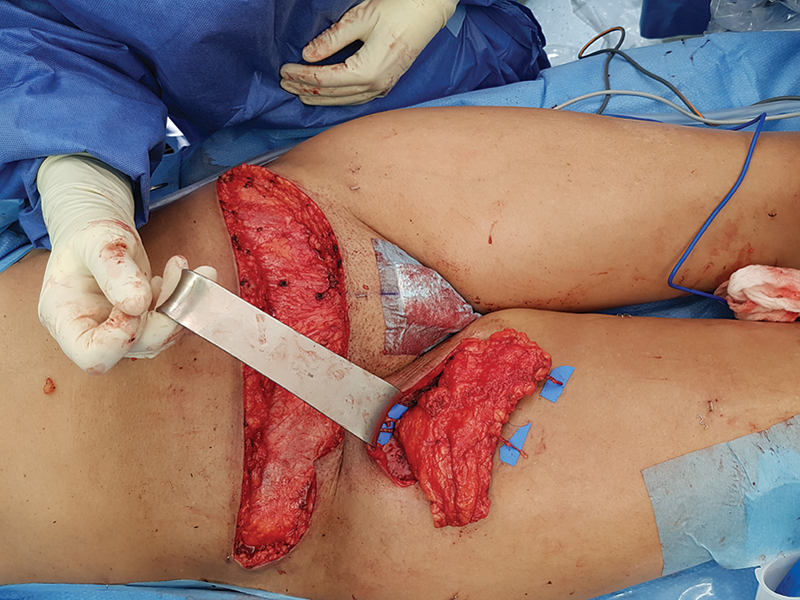
Flap inset with positioning of recipient veins.

**Fig. 4:**
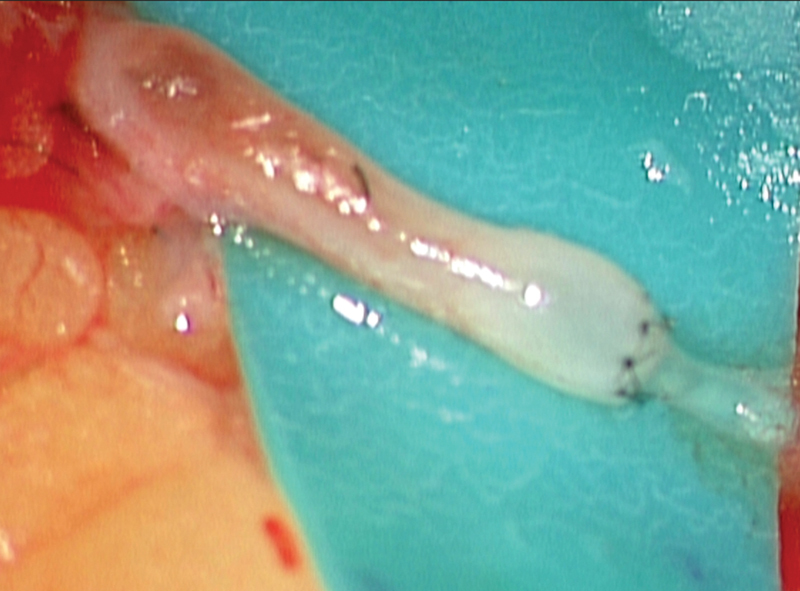
Intraoperative view of lymphovenular anastomosis.

The pinch test allowed to design a mini-abdominoplasty SIEA flap with adequate dimension to obliterate the dead space of the groin (the flap was tailored on the defect 16 cm × 6 cm). The pedicled flap was harvested preserving two superficial veins (draining on the SIEV system) on the cranial border of the flap. The whole flap was deepithelialized leaving just a small skin island for postoperative monitoring. The flap was transposed and twisted to fill the defect, then two LVAs were performed between the two veins and two lymphatics, and the remaining third lymphatic was clipped ( Figs. 3 and 4 ). After confirming adequate flap perfusion with ICG, all the wounds were closed.

**Fig. 5:**
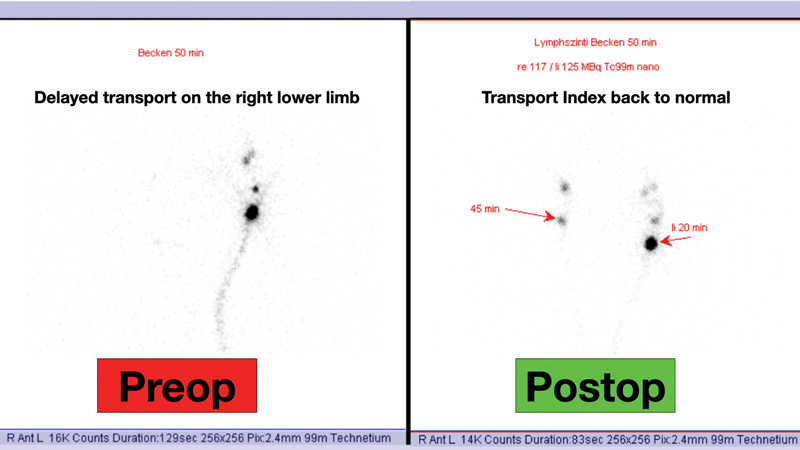
Postoperative lymphoscintigraphy, the transport index changed from 15 preoperatively to 1.8 postoperatively.

**Fig. 6:**
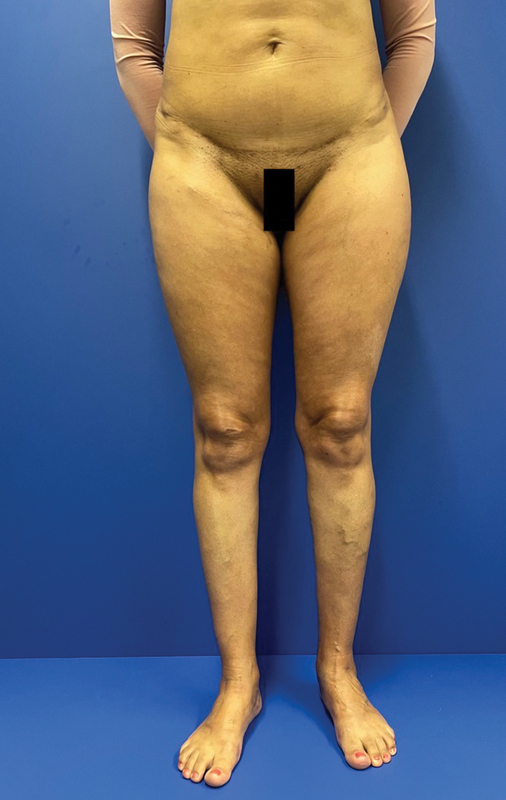
Final result 1 year after surgery with removal of flap monitoring skin paddle.

The postoperative course was uneventful with resolution of the lymphocele. The lymphoscintigraphy performed 3 months after surgery showed the resolution of the lymphocele and a normalized Transport Index (from 15 to 1.8 13 ; Fig. 5 ). The monitor skin island was removed under local anesthesia 2 months afterwards with optimal aesthetic contour ( Fig. 6 ).

The patient signed a written informed consent for the procedure and to be included in the study in accordance with the Second Helsinki Declaration.

### Literature Review

The literature in the Medline database (PubMed) was searched using combinations of key words (“recurrent lymphocele,” “groin lymphocele,” “lymphocele of the lower limb,” “groin lymphorrhea,” “groin lymphatic fistulas”). Studies published in English describing the management of lymphocele with LVAs or flaps were selected. Of the over 400 papers that deal with the treatment of recurrent lymphocele, 9 articles (39 patients) respected the inclusion criteria. No cases of groin lymphocele treated with flaps alone have been identified. A case of pelvic lymphocele successfully treated with a pedicled deep inferior epigastric rerforator (DIEP) flap after LVA has been reported 14 ( Table 1 ).

**Table 1:** Literature review of cases of recurrent lymphocele of the groin managed with flap or lymphovenular anastomosis

	Author and year	Number of cases successfully treated
**LVAs**	Boccardo et al 2014 15	16
	Yamamoto et al 2014 16	3 LVA and 1 LLA
	Ayestaray et al 2017 17	CR
	Giacalone et al 2019 18	1 case in the groin and 3 in the leg
	Shimono et al 2020 19	11
	Gabriele et al 2020 20	CR
	Scaglioni et al 2021 21	CR
	Kadota et al 2021 22	3 cases in the groin
	Mitsui et al 2022 8	CR
**Trans-flap LVAs**	Guillier et al 2022 23	4 with lymphorrea and 1 with lymphedema

Abbreviations: CR, case report; LLA, lymphaticolymphatic anastomosis; LVA, lymphovenular anastomosis; LyFT, lymphatic flow-through.

## Discussion

The ideal treatment of lymphocele has not been established. Conservative treatments, have been used, 2 however, intractable cases may recur also after lymphatic embolization and microscopic ligation of lymphatic vessels. Selective ligation of the leaking lymphatic vessels could be considered when supported by lymphatic mapping. 24 25 26 27 However, previous systematic reviews were not able to identify the best treatment for lymphatic fistulas, including muscle flaps. 28 29 Nowadays, complete capsule excision and LVAs are considered the most appropriate therapeutic approach for lymphedema and showed promising results in the treatment of recurrent lymphoceles limiting lymph flow in the cyst of the lower limb. 10 18 22 Similarly, lymphaticolymphatic anastomosis have been described. 16

The lack of suitable recipient veins to perform the anastomosis is a well-known problem in the clinical setting. Good caliber match, adequate position related to the lymphatic vessel, and good quality of the recipient vein are three characteristics that rarely come together in the operative field, especially at the sites of previous surgery or in patients with a history of venous diseases. The possibility of performing additional LVAs on recipient veins, deep 9 11 or superficial, 12 provided by the transposition of a flap overcomes the lack of suitable veins in patients with groin defects with injured collectors. Both recipient sites have shown effective results, even if subdermal venules with intact valves are usually recommended for anastomosis due to their small diameter and lower intravenous pressures.

Initially presented as a strategy to prevent lymphedema or lymphatic complications during primary cancer surgery, 9 30 31 32 33 34 trans-flap LVAs have recently proposed for soft tissue reconstruction and treatment of lymphatic leakage and lower limb lymphedema. 23 Some surgeons may argue with the application of the flow-through terminology to the lymphatic deviation within the flap. We believe that such definition as previously published is intuitive, however, we agree that the lack of a proximal lymph vessel makes it debatable.

The early resolution of the lymphatic overflow and lymphocele in our case supports the role of LVAs in the restoration of lymphatic drainage.

As for the choice of donor sites, the reconstructive surgeon should always aim to restore the form and function of the affected area with minimal secondary damage. The abdomen as a donor site has unique advantages in terms of tissue bulk, cosmetics, and reach. Thus, it should be considered a valuable tool for the reconstruction of the groin region.

A large SCIP flap (required to obliterate the dead space) may be associated with a higher scar and an asymmetric pull of the umbilicus. The ALT flap can be considered a workhorse flap for groin reconstruction, however, the scar in the thigh area is not as concealable as the mini-abdominoplasty-like scar. The amount of abdominal tissue to be used can be determined by pinch test in such a way to be sure to obtain complete filling of the dead space and avoid unpleasant pull/distortion or visible scarring on the abdomen.

The SIE artery originates from the femoral triangle and travels in the subcutaneous tissue over the inguinal ligament. It vascularizes mostly the inferomedial part of the abdomen. A flap harvested on the SIEA does not require intramuscular dissection, the course of the artery provides a convenient pivot point to reach the inguinal and proximal-thigh regions. Superficial veins of the abdomen drain in the SIEV system which can be preserved within the pedicled fashion flap.

When the vascular anatomy of the SIEA is questionable, a similar flap with cranial LVAs' recipient veins but based on deep inferior epigastric artery or superficial circumflex iliac artery systems could be planned but the risk of abdominal wall weakening should be discussed with the patient in case of DIEP flap harvest. 35

The use of the abdominal flaps allowed the simultaneous restoration of the body contour and the reestablishment of lymphatic drainage. In fact, abdominal flaps minimize the scar burden compared with alternative donor area.

It is still under debate if the additional intraflap LVAs are the key factor to restore the lymphatic channels or if the flap itself would have been enough as postulated with the LIFT concept. However, the latter has been applied as preventive strategy and its effect in recurrent lymphocele has not been reported yet. 36 Further studies are needed. We believe that the early restoration of the lymphatic drainage may be considered a signal to support the benefit of lymphatic flow-through anastomosis.

The present report seems to confirm the benefit of using distal veins of a flap as recipient for LVAs for the treatment of condition related to interrupted lymphatic pathways such as lymphoceles. Given the functional restoration and good aesthetic outcome, LVAs in a pedicled SIEA flap can be considered a tool to restore the lymphatic drainage of the groin.
